# Dengue virus NS1 protein does not activate TLR4 and has a modest effect on endothelial monolayer integrity compared to TNF

**DOI:** 10.1371/journal.ppat.1013695

**Published:** 2025-11-11

**Authors:** Parimala R. Vajjhala, Jaehyeon Kim, Adriana Pliego Zamora, Helen Mostafavi, Larisa I. Labzin, Katryn J. Stacey

**Affiliations:** 1 School of Chemistry and Molecular Biosciences and Australian Infectious Diseases Research Centre, The University of Queensland, Brisbane, Queensland, Australia; 2 Institute for Molecular Bioscience, The University of Queensland, Brisbane, Queensland, Australia; SUNY Upstate Medical University: State University of New York Upstate Medical University, UNITED STATES OF AMERICA

## Abstract

Severe dengue virus (DENV) infection is characterised by vascular leak and can lead to shock and death. The virus-derived non-structural protein 1 (NS1), which is released into the circulation as a lipoparticle, has been implicated in exacerbation of inflammation through activation of Toll-like receptor 4 (TLR4), and in disruption of endothelial monolayer integrity. We sought to identify the component of the NS1 lipoparticle that activates TLR4. Native and C-terminally His-tagged recombinant NS1 were produced in suspension HEK293 cells and formed hexamers indicating structural integrity. However, neither the recombinant NS1 nor NS1 secreted by DENV-infected Vero or K562 cells activated TLR4. Thus, the NS1 apoprotein itself does not activate TLR4, although it could potentially associate with inflammatory lipids as a lipoparticle. Notably, the lack of TLR4 stimulation by NS1 protein does not exclude a role for TLR4 in dengue disease as it may be activated by LPS influx from the gut or by putative endogenous stimulatory lipids. Furthermore, purified His-tagged NS1, at a biologically relevant concentration, only had a modest effect on endothelial monolayer integrity as assessed by changes in the electrical impedance of an endothelial monolayer. In contrast TNF, a cytokine with an established role in vascular leak, had a potent effect when used at a concentration seen in severe dengue disease. Medium from DENV-infected human macrophages also had a potent disruptive effect. This medium had low NS1, compared to that encountered in some patients, but high TNF concentration and its effect on endothelial monolayers was mostly ablated by an anti-TNF antibody. While multiple factors are likely to contribute to DENV-associated vascular leak in vivo, our data support a key role for TNF. Importantly, our data indicate that while NS1 has a modest effect on endothelial monolayer integrity there is no evidence for it activating TLR4.

## Author summary

Dengue virus is a mosquito-borne flavivirus that causes a range of symptoms ranging from mild fever to severe disease. Severe disease is characterised by leak of plasma from the blood vessels that can lead to life-threatening shock. Factors implicated in severe disease include proinflammatory cytokines as well as the dengue virus non-structural protein 1 (NS1), which is secreted by infected cells. Previous reports suggested that NS1 could induce inflammation in the same way as bacterial lipopolysaccharide (LPS) which acts through Toll-like receptor 4 (TLR4). Here we show that dengue NS1 produced in mammalian cells does not activate TLR4. However, results from a cell culture model system suggest that NS1 may still modestly induce blood vessel leak. This was much less marked than for the inflammatory cytokine TNF that is known to mediate blood vessel permeability. Our results are important for understanding the causes of severe dengue disease and development of therapeutic agents.

## Introduction

Dengue virus (DENV) causes a spectrum of diseases ranging from mild dengue fever (DF) to the severe dengue haemorrhagic fever (DHF) and dengue shock syndrome (DSS), which are characterised by vascular permeability [1,2]. There are an estimated 390 million infections from this mosquito-borne flavivirus each year, largely in the tropics, with increasing infections attributed to factors such as climate change and urbanization [1,3]. There are no specific treatments for severe DENV infection, and thus identifying the key molecules that drive vascular leak associated with severe disease is critical for development of therapeutic strategies. Severe disease may emerge after 3–6 days of symptoms, when patients develop vascular leak and internal haemorrhage at a time when viremia and fever are abating [2,4]. There are four serotypes of the virus and severe disease cases are frequently but not exclusively caused by secondary infection with a different serotype. Cross reactive non-neutralising antibodies are suggested to increase viral uptake via Fc receptors, a phenomenon termed antibody-dependent enhancement (ADE) of infection [5]. While this leads to high viremia, this does not appear to be the sole cause of severe disease [2], with host genetics and the virus strain also playing a role [6].

There are a number of host-derived molecules proposed to mediate DENV-induced vascular leak including cytokines such as TNF, IL-6, IL-1β, CXCL8, MIF, CCL2, VEGF, inflammatory lipid mediators such as PAF and leukotrienes, as well as matrix metalloproteinases and glycosaminoglycans [2,7]. The critical role of TNF in mouse models of severe DENV infection has been well established with an anti-TNF antibody inhibiting vascular leak and promoting survival of infected mice [8–10]. Autopsy samples from DENV patients have shown higher levels of TNF in liver, lung and kidneys compared to samples from non-DENV infected patients [11] and some studies have shown high circulating TNF in patients with severe disease compared to mild DF [12–15]. However, other studies have not shown a correlation with severity [16]. In addition, bacterial lipopolysaccharide (LPS), a well-known mediator of vascular leak [17], can be detected in the blood of patients and levels correlate with disease severity [18]. We recently showed that the gastrointestinal tract is the major site of observable inflammation and pathology in DENV-infected mice [19]. Consequently, gut damage may explain elevated circulating LPS. In agreement with this, severe DENV infection in patients is frequently accompanied by gastrointestinal symptoms including abdominal pain and vomiting [2]. Finally, there is considerable evidence indicating a role for the DENV non-structural protein 1 (NS1) in mediating severe dengue disease [20–23].

NS1 is the only viral protein that is secreted during DENV infection and a correlation between severe DHF and high NS1 levels has been shown [24]. NS1 levels in DHF were mainly between 0.6 and 2.5 μg/mL in one study [24] but levels as high as 50 μg/mL have been reported [25]. However, not all patients with high NS1 develop severe disease [24] and data from mouse models show that NS1 levels do not always correlate with disease severity [26]. NS1 initially forms a membrane-bound dimer in the ER and is required for viral replication [27], but can also traffic to the cell surface and be released into circulation. NS1 purified from the medium of infected Vero cells or upon recombinant expression and secretion from HEK293 or S2 insect cells revealed a hexameric open-barrel structure with a lipid core [28–30]. NS1 tetramer and dimer forms are also detected in recombinant NS1 expressed in suspension HEK293 cells [31]. However, recent studies suggest that NS1 secreted from infected Vero cells or from DENV-infected mouse or human sera is a dimer associated with serum high density lipoprotein (HDL) [32]. Apolipoprotein (apo) A-I, the major protein of HDL, interacts with NS1 [33], and is suggested to dissociate the NS1 hexamer and allow NS1 dimer binding to HDL and to a lesser extent to low density lipoprotein (LDL) [34]. Previous studies have suggested that recombinant DENV serotype 2 (DENV2) NS1 mediates proinflammatory effects via TLR4 [21,33,35], best known for its recognition of lipopolysaccharide (LPS) from Gram-negative bacteria [36]. Importantly, recombinant DENV2 NS1 has been shown to disrupt endothelial monolayer integrity [20,21], which was suggested to be TLR4-dependent [21] or via damage to the glycocalyx involving sialidase activation [22]. Furthermore, NS1-mediated endothelial permeability is proposed to aid in dissemination of the virus [37]. Thus, NS1 is implicated in the development of severe dengue disease.

Here we found that DENV2 NS1, either recombinantly expressed in mammalian cells or secreted from DENV-infected cells, failed to activate TLR4. This indicates that the NS1 apoprotein lacks TLR4-stimulatory activity, and prior results may have been due to a TLR4-stimulatory lipid component associated with the NS1 lipoprotein particle. In this study, purified NS1 had a modest effect on endothelial monolayer integrity in vitro compared to purified TNF when both were tested at clinically relevant concentrations. Furthermore, medium from DENV-infected macrophages potently disrupted monolayers, in a TNF-dependent manner. Our data support a key role for TNF in DENV-induced vascular leak, with a lesser direct effect of NS1 on endothelial cells.

## Results

### Untagged NS1 expressed in mammalian cells forms hexamers but does not activate TLR4

**Fig 1:**
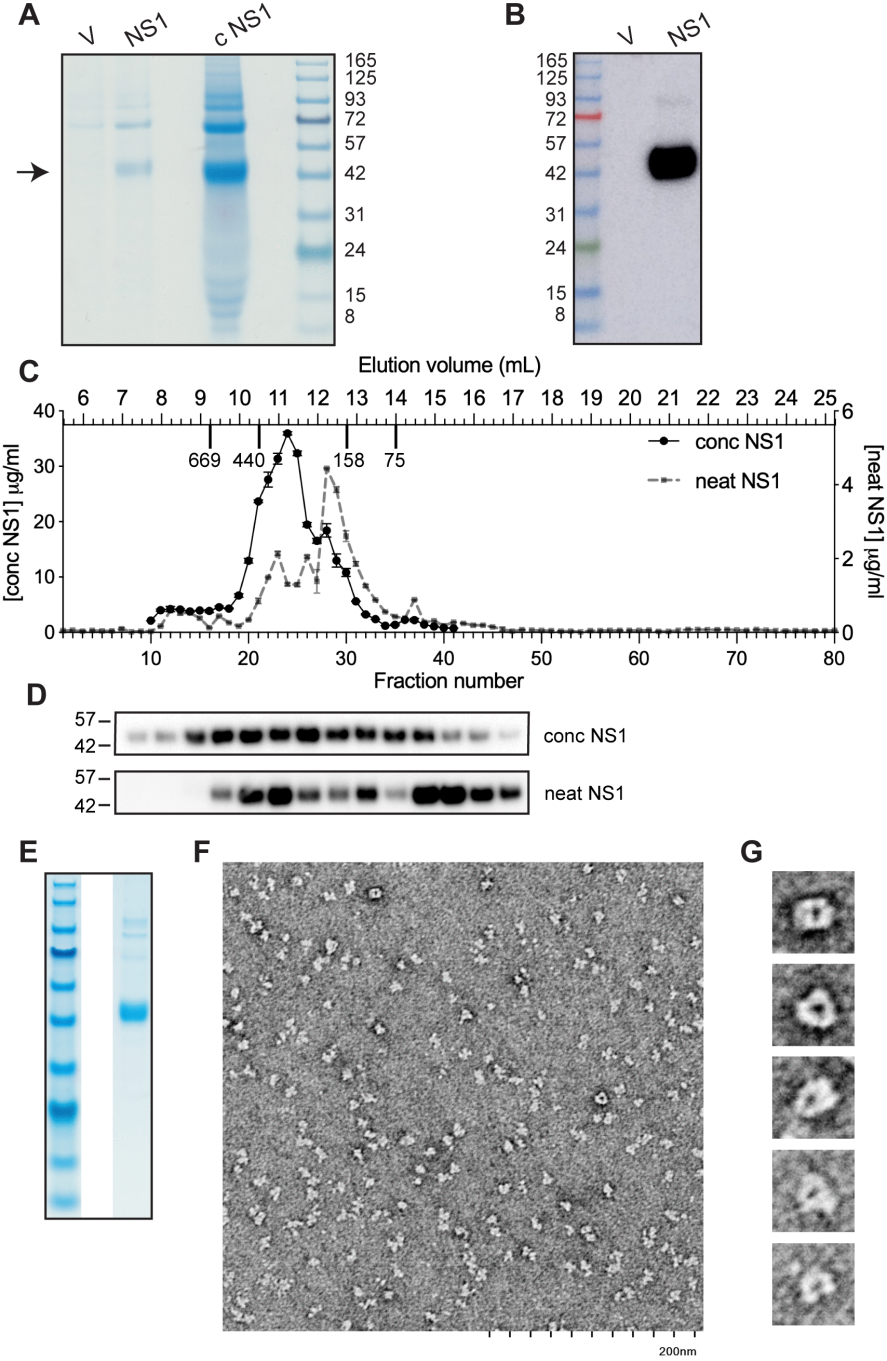
NS1 expressed in suspension HEK293 cells can form hexameric complexes. A. SDS-PAGE and Coomassie stain of medium from HEK293 suspension cells transfected with empty vector (V) or NS1 expression plasmid. NS1 expression medium before (NS1) and after concentration (cNS1) are shown, with respective loading of approximately 0.5 µg and 5 µg NS1, as assessed by ELISA. Arrow indicates the position of the NS1 protein. B. Immunoblot analysis of neat medium with anti-NS1 antibody (1H7). In A and B, 10 μL of sample was loaded on the SDS gels and 3 μl of marker, which contains 0.2-0.4 μg/μL of each protein. C. Size exclusion chromatography of neat or concentrated NS1 expression medium. Sample (0.5 mL) was loaded on the column and 0.25 mL fractions collected. The level of NS1 in the fractions was determined by ELISA. The elution volumes and molecular weights of proteins used to calibrate the column, as determined by the manufacturer, are indicated on the upper X-axis. D. Immunoblot analysis of selected NS1-containing fractions, 18-31, from SEC. E and F, Fractions 22-25 from SEC of concentrated NS1 were pooled and concentrated 20-fold on a 100 kDa cut-off filter and then aliquots were analysed by SDS-PAGE and Coomassie stain (E) and by negative stain EM (F). G. Enlargement of selected particles from EM showing ring-shaped oligomers.

We initially aimed to check the activation of TLR4 by NS1 using the secreted recombinant protein without purification in order to eliminate the possibility of LPS contamination. NS1 was expressed as a native protein with an IgK signal peptide in suspension HEK293 cells. The NS1 concentration estimated by ELISA in expression medium directly harvested from cells, referred to as “neat” NS1, was as high as 55 μg/mL. After concentration approximately 10-fold on a 10 kDa cut-off centrifugal filter unit, the NS1 concentration was 520 μg/mL. SDS-PAGE and Coomassie stain indicated that the NS1 expression medium contained an abundant protein with a molecular weight of 45 kDa, not present in the vector alone control (Fig 1A). This is consistent with the previously observed molecular weight range of 46–55 kDa for NS1, which is attributed to variable glycosylation [27]. Furthermore, the main protein in the expression medium was verified to have the same molecular weight as NS1 by western blotting (Fig 1B).

To establish whether the NS1 protein was in a native hexameric state, the expression medium containing NS1 was analysed by size exclusion chromatography (SEC), using both the neat and concentrated media. Analysis of the SEC fractions by NS1 ELISA indicated that the NS1 in the concentrated expression medium predominantly eluted in a major peak with indicative molecular weight of 266–422 kDa (Fig 1C). This is comparable to the molecular weight range previously reported for purified insect cell-expressed NS1 hexamer, which was 250–350 kDa [29]. In contrast a smaller fraction of the NS1 in the neat medium eluted at this high molecular weight, while the bulk of it eluted with a molecular weight of 154–194 kDa, which is likely a combination of tetramer and dimer forms [31]. Peak NS1 fractions were analysed by immunoblot analysis (Fig 1D) and this verified that the bulk NS1 in the concentrated sample was present in fractions consistent with hexamer formation while that in the neat medium was split between likely hexamer and the lower molecular weight tetramer/dimer states.

To confirm that the early eluting fractions of NS1 contained discrete hexamers, protein in those fractions was analysed by electron microscopy. Fractions 22–25 from SEC of concentrated NS1 medium were pooled and concentrated on a 100 kDa molecular weight cut-off filter unit, with NS1 protein accounting for 84% of the total protein based on densitometry upon SDS-PAGE (Fig 1E). Analysis by negative stain electron microscopy indicated the presence of discrete particles that were fairly uniform in size (Fig 1F). Particles in the appropriate orientation indicated a ring-shaped structure (Fig 1G) with a diameter of 10 nm in agreement with structural studies that have shown that the NS1 hexamer forms a barrel-shaped protein shell with a 10 nm diameter, which surrounds a lipid core [28–30].

**Fig 2:**
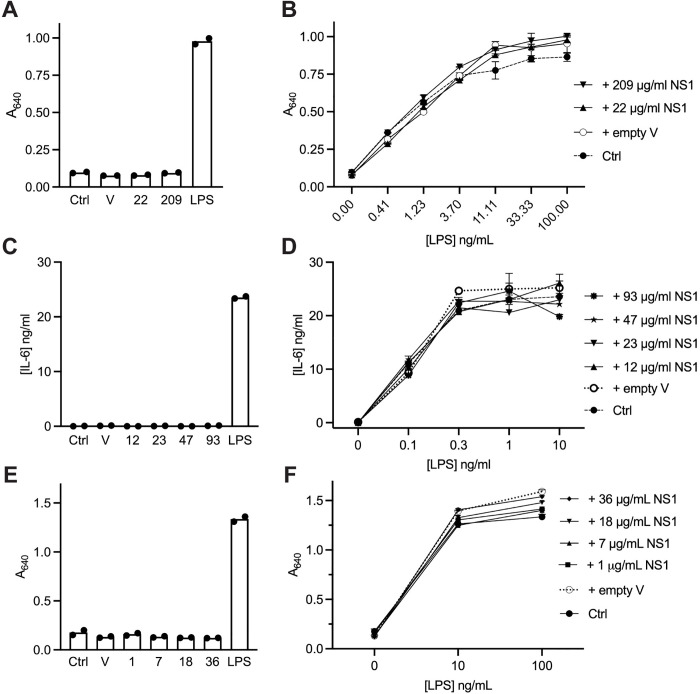
NS1 expressed in suspension HEK293 or CHO-S cells does not activate TLR4. HEK-Blue hTLR4 cells (A, B, E and F) were treated with neat or concentrated NS1 expression medium or empty vector medium (V) from suspension HEK293 cells (A and B) or CHO-S cells (E and F) or with DMEM medium (Ctrl) alone or in the presence of LPS. PBMC (C and D) were treated with buffer-exchanged NS1 expression medium or empty vector medium (V) from suspension HEK cells, or with RPMI medium (Ctrl) alone or in the presence of LPS. A, C and E are selected samples from B, D and F to show the response of cells treated with NS1 alone, empty vector medium alone, 100 ng/ml LPS alone, or cell culture medium alone. B, D and F show dose responses of the cells to LPS in the absence (Ctrl) or presence of NS1 at the different concentrations or in the presence of empty vector medium. The final concentration of NS1 is stated. For assays with HEK-Blue hTLR4 cells, the A_640_ measured in the SEAP reporter assay is presented. For PBMC, IL-6 released into the medium after 18 h, detected by ELISA, is presented. Data in A-D are representative of two independent experiments using two independent expression media samples. Data from E and F are from one experiment. Error bars represent the range of 2 replicates.

To prevent any contamination of the NS1 with LPS, which could occur during purification and handling, the expression medium with secreted recombinant untagged NS1 was used directly or after concentration using a 10 kDa cut-off centrifugal filter unit to test TLR4 activation in HEK-Blue human TLR4 (hTLR4) cells. In these cells, TLR4 signalling drives an NF-κB-driven secreted embryonic alkaline phosphatase (SEAP) reporter, which is assayed. We treated the cells with HEK293-expressed NS1 at concentrations of 22 or 209 μg/mL, which were equivalent to, or 10-fold higher than previous studies [21,35]. There was no activation of TLR4 at these concentrations of NS1 (Fig 2A). To test whether there was anything in the NS1 expression medium that inhibited activation of TLR4, LPS was added to the cells together with the NS1 medium, however, no inhibition of the response to LPS was observed (Fig 2B). There was a slight increase in the TLR4 response to LPS with NS1 medium compared to the control but this was similar to the response to LPS with the empty vector control medium indicating that the increase was not due to NS1. We also treated peripheral blood mononuclear cells (PBMCs) with the HEK293-expressed NS1. The NS1 medium and empty vector medium were first buffer exchanged into PBS and then used to treat PBMCs. No release of the IL-6 cytokine was detected from PBMCs treated with NS1 at concentrations ranging from 12 to 93 μg/mL (Fig 2C), which is equivalent to or higher than the concentrations used in the previous study [21]. Furthermore, the NS1 samples did not affect the response of the PBMCs to LPS (Fig 2D). Finally, we tested neat and concentrated medium from CHO-S cells transfected with either a plasmid expressing NS1 or empty vector and found that the NS1 produced by these cells also failed to activate HEK-Blue hTLR4 cells when tested at concentrations of up to 36 μg/mL (Fig 2E) and had no inhibitory effect on LPS response when compared to medium from empty vector-transfected CHO-S cells (Fig 2F). Thus, we conclude that the recombinant NS1 expressed from suspension HEK293 and CHO-S cells under current conditions does not activate TLR4.

### NS1 released by DENV-infected Vero and K562 cells does not activate TLR4

**Fig 3:**
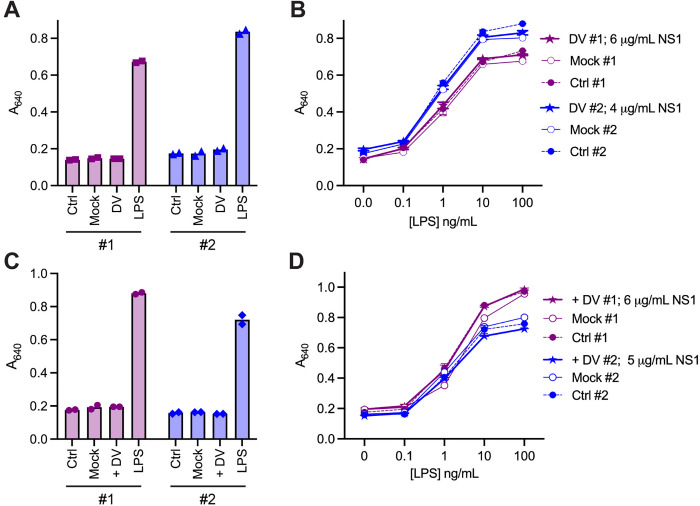
Media from DENV-infected Vero or K562 cells, which contain NS1, do not activate TLR4 or potentiate the TLR4 response to LPS. HEK-Blue hTLR4 cells were treated with buffer-exchanged, concentrated control sterile medium (Ctrl) or media from mock or DENV (DV)-infected Vero (A and B) or K562 cells (C and D). Treatment was alone or in the presence of LPS. Data in A and C show selected samples from B and D to show the TLR4 response to the different media in the absence of LPS or to LPS alone (10 ng/mL). Data shown are from two independent experiments [# 1 and # 2] each using media from independent infections. In A and B, media harvested on day 4 were used. In C and D media harvested on day 4 [# 1] or day 7 [# 2] from independent infections were used. Final NS1 concentrations are stated in the legends for B and D. Error bars on B and D represent the range of duplicate wells, but are mostly smaller than the symbol.

Since we showed that recombinant NS1 does not activate TLR4, a key question was whether NS1 released from DENV-infected cells, which is likely to resemble the NS1 produced during dengue disease, can activate TLR4. To generate virally-produced NS1 we infected Vero cells, which are kidney epithelial cells from an African green monkey, as the NS1 hexamer released by these cells has been structurally well characterised [28,32] as well as human K562 cells. The media from infected or mock-treated cells as well as control sterile media were buffer exchanged into PBS and concentrated prior to assessing TLR4 activation on HEK-Blue hTLR4 cells. However, no TLR4 activation was observed with media from infected Vero (Fig 3A and 3B) or K562 cells (Fig 3C and 3D) containing 4–6 μg/mL NS1. This is comparable to the concentration of CHO-S cell-expressed NS1 (5 μg/mL) previously shown to activate TLR4 [21]. Furthermore, medium from infected cells did not potentiate or inhibit the TLR4 response to LPS (Fig 3B and 3D). We conclude that the NS1 produced by DENV-infected cells, like the recombinant NS1, does not activate TLR4.

### His-tagged NS1 does not activate TLR4 but has a minor effect on HUVEC monolayer integrity

**Fig 4:**
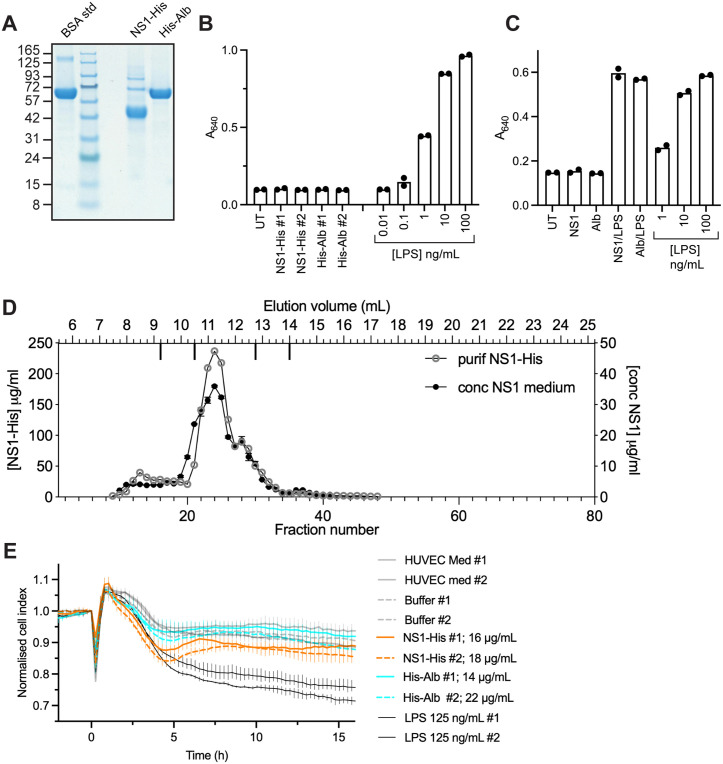
Purified His-tagged NS1 does not activate TLR4 but has a modest effect on endothelial monolayer integrity. A. SDS-PAGE and Coomassie stain of purified, buffer exchanged, His-tagged NS1 and albumin. Samples were 5 μg of BSA protein standard and 4 μL of purified protein containing 3.5 μg or 2.8 μg of His-tagged albumin or NS1 respectively. B. Treatment of HEK-Blue hTLR4 cells with purified His-tagged NS1. Cells were treated with two independently purified samples of NS1 (31 and 37 μg/mL) or albumin (27 and 46 μg/mL) or with LPS at stated concentrations (ng/ml). Two replicate wells were treated with each sample. C. Treatment of HEK-Blue hTLR4 cells with purified His-tagged NS1 in the presence or absence of LPS. HEK-Blue hTLR4 cells were treated with NS1 (59 μg/mL) or albumin (52 μg/mL) with or without LPS at 100 ng/mL. D. Size exclusion chromatography of purified His-tagged NS1. Sample (0.5 mL) was loaded on the column and 0.25 mL fractions collected and analyzed as in Fig 1C. The chromatogram for concentrated untagged NS1 from Fig 1C is superimposed for comparison. E. NS1 has a modest effect on endothelial monolayer integrity. HUVEC cells were plated in a 96-well E-plate and treated with two independent samples of batch purified and buffer exchanged NS1 or albumin at the stated concentrations or with LPS. Changes in monolayer integrity were assessed using an xCELLigence RTCA instrument. Each line represents the mean of three technical replicates except for HUVEC medium #2, which has two replicates, and error bars show the range. The data are representative of at least 4 independent experiments done using NS1 at concentrations ranging from 12-35 μg/mL, each experiment with 2-3 replicates.

Since previous work has shown that NS1 disrupts endothelial monolayer integrity [20–22,38], we assessed whether the NS1 protein without associated TLR4-activating material behaves similarly. Albumin was produced in parallel to show that any effects on monolayer integrity were specific to NS1 and not due to contaminants acquired during expression or purification. Both His-tagged proteins were expressed in suspension HEK293 and purified (Fig 4A). Neither of the purified proteins activated HEK-Blue hTLR4 cells (Fig 4B), which agrees with the data for untagged NS1 (Fig 2) and is expected for albumin. Furthermore, neither of the proteins had any effect on the TLR4 response to LPS (Fig 4C). Finally, analytical size exclusion chromatography showed that His-tagged NS1 mainly formed a hexamer similar to the untagged NS1 (Fig 4D).

To assess the effect of NS1 on endothelial monolayer integrity we used the xCELLigence RTCA instrument that monitors the electrical impedance of cell monolayers by measuring the electrical current between gold microelectrodes at the bottom of a 96-well plate, with the output expressed as a normalised “cell index”. This instrument has been used to study changes in the monolayer integrity of endothelial cells [39–43]. Human umbilical vein endothelial cells (HUVEC) were plated and grown for 3 days allowing a monolayer to form with a plateau in the cell index, before treatment. Upon addition of any sample to the monolayer, including medium alone, there is always an immediate transient perturbation in cell index associated with temperature fluctuation and new medium (Fig 4E). Following the addition of albumin, the cell index remained very similar to cells treated with HUVEC medium alone or the buffer control. However, upon addition of NS1 a slight decrease in cell index with a nadir at ~5 h was consistently observed, which reflects a decrease in monolayer integrity (Fig 4E). This effect was minor when compared to that of LPS at 125 ng/mL, which is well known to disrupt endothelial monolayer integrity [17]. We conclude that the purified NS1 has a slight effect on endothelial monolayer integrity.

### Medium from DENV-infected macrophages has a potent effect on endothelial monolayer integrity

**Fig 5:**
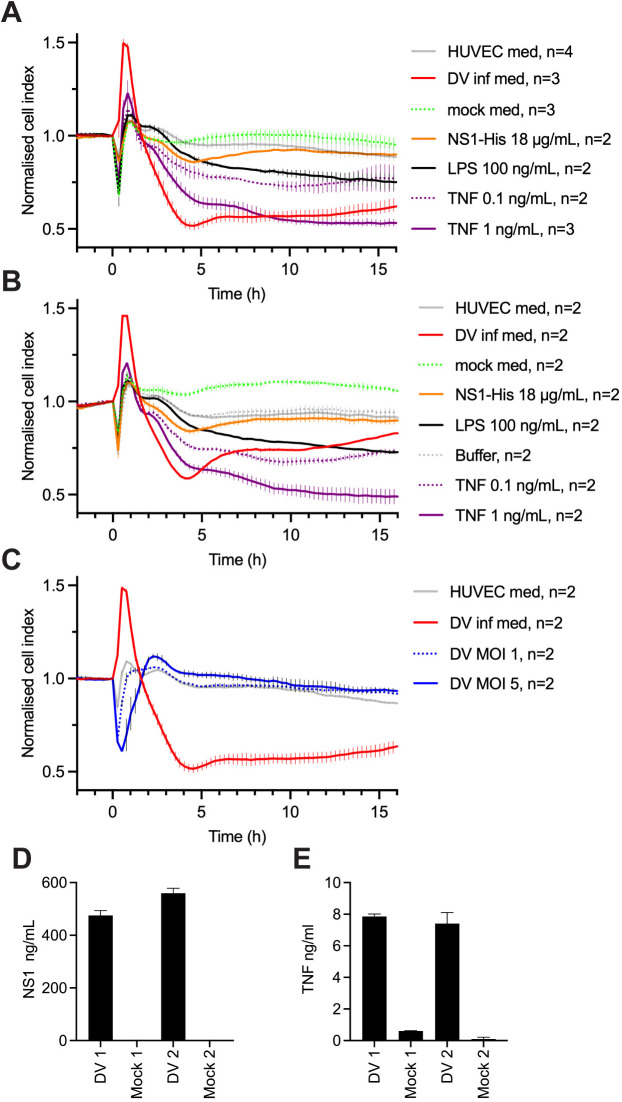
Medium from DENV infected cells has a profound effect on endothelial monolayer integrity. A and B, HUVEC cells plated in a 96-well E-plate were treated with two independent samples of medium from GM-CSF macrophages infected with DENV (DV med) or mock-infected (mock med) and various control samples. Each panel shows data generated using a single sample of medium from DENV-infected or mock-infected macrophages. Changes in monolayer integrity were assessed using the xCELLigence RTCA instrument. In A, error bars reflect the range of replicates, which were spread between two experiments except for NS1, which was all in the second experiment. In B, error bars reflect the range of 2 replicates all from the same experiment. C. HUVEC cells plated in a 96-well E-plate were treated with DENV at MOI 1 or 5. Also shown for comparison are untreated cells (HUVEC medium), and cells treated with 40 μL of DENV-infected medium, which were in the same experiment, but which are also shown in panel A. Error bars represent range of two replicates all in the same experiment. D. NS1 concentrations in media from DENV-infected and mock-infected GM-CSF macrophages measured by NS1 ELISA. E. TNF concentrations in media from DENV- and mock-infected GM-CSF macrophages measured by AlphaLISA.

It was unclear how significant the effect of NS1 is on endothelial cells when compared to other factors released by DENV-infected cells. Since myeloid cells including dendritic cells, monocytes and macrophages are the major cells infected *in vivo* by DENV [44], we aimed to compare the effect of purified NS1 on endothelial monolayer integrity with that of the medium released from DENV-infected GM-CSF-differentiated macrophages. When medium from DENV-infected macrophages was diluted 5-fold into the wells with HUVEC monolayers, it elicited a far more profound disruptive effect on monolayer integrity than that elicited by purified NS1 (Fig 5A). When compared with TNF, which is implicated in vascular leak in mouse models of severe DENV infection [8–10], the effect of the diluted medium was comparable to 1 ng/mL TNF. Notably, the effect of purified NS1 was markedly less than the effect of 0.1 ng/mL TNF at time points later than 5 h. There was no effect of medium from mock-infected macrophages compared to control HUVEC medium. A replicate experiment gave very similar results for the comparison of the effect of virus-infected and mock-infected medium. However, in this case the medium from the mock-infected cells appeared to increase cell index (monolayer integrity) when compared to untreated cells (Fig 5B), which we believed was due to both virus- and mock-infected media samples being more visibly acidified than in the previous experiment. Notably, when the HUVEC monolayers were treated with DENV at MOI 1 or 5, the virus alone did not disrupt monolayer integrity over this time course (Fig 5C).

Previous studies have shown that DENV-infected GM-CSF macrophages produce high levels of TNF and that the medium can mediate endothelial permeability as measured by permeability of a HMEC-1 endothelial monolayer to horseradish peroxidase protein [45]. However, the level of NS1 was not measured and the relative contribution of TNF and NS1 to endothelial permeability was not assessed. Here, the concentration of NS1 in the media from infected macrophages was 475 or 560 ng/mL for the two different samples (Fig 5D), thus when diluted 5-fold into the wells with HUVEC cells the concentration was ~ 100 ng/mL. This is more than 160-fold less than the concentration of purified NS1 added to the HUVEC cells in Figs 4E, 5A and 5B (16–18 μg/mL). Thus, it is unlikely that the NS1 in the media from infected macrophages is playing a key role in disrupting endothelial integrity in these experiments. The concentrations of TNF measured in the media from infected macrophages were 7 and 8 ng/mL (Fig 5E), thus the final concentrations of TNF after a 5-fold dilution of the media in the wells with HUVECs would be 1.4 or 1.6 ng/mL, respectively. The effect of the infected medium was comparable with the effect of 1 ng/mL of TNF (Fig 5A) suggesting that TNF produced by the macrophages is likely to be a major mediator of the observed endothelial monolayer disruption.

### Anti-TNF antibody largely ablated the effect of the DENV-infected macrophage medium on endothelial monolayer integrity

**Fig 6:**
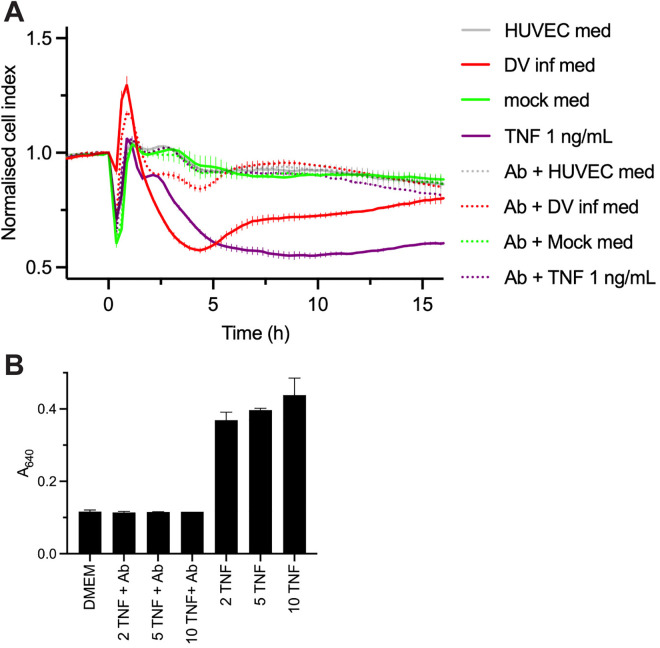
TNF is the key factor in medium from DENV-infected GM-CSF macrophages that disrupts endothelial monolayer integrity. HUVEC cells were plated in a 96-well E-plate and treated with media from DENV- or mock-infected GM-CSF macrophages which were preincubated with or without anti-TNF antibody (Ab). In A, error bars represent the range of two replicates, all in the same experiment. B. TNF samples at 20-100 ng/mL were preincubated with anti-TNF Ab at 100 μg/mL and then diluted ten-fold into wells with HEK Blue hTLR4 cells for 18 h. The A_640_ measured in the SEAP reporter assay is presented. In B, error bars represent the range of duplicate wells, except for the sample with 10 ng/mL TNF + Ab, which was from a single well.

When medium from the DENV-infected macrophages was pre-incubated with a blocking antibody against human TNF, its ability to disrupt HUVEC monolayer integrity was largely ablated (Fig 6A). Preincubation of anti-TNF with 1 ng/mL TNF completely ablated the effect of TNF, returning it to the same level as untreated cells with HUVEC medium alone. The anti-TNF antibody alone had no effect on untreated cells or mock-infected medium. We confirmed that at the concentration used (10 µg/ml), anti-TNF can neutralise TNF at up to 10 ng/mL, when tested on HEK-Blue Null1 cells that lack TLR4 but respond to TNF (Fig 6B). Since the concentration of TNF from the DENV-infected medium was 0.7 ng/mL after adding to the HUVEC in Fig 6A, it is likely that all the TNF was neutralised and the residual effect on endothelial monolayer integrity is due to other factors. A contribution to the residual effect by NS1 secreted by the macrophages is likely to be minor since it is at a concentration more than 160-fold less than the purified NS1 used in Figs 4E, 5A and 5B, which only had a modest effect.

The viral titre of the DENV-infected medium was assessed at the same time it was used in the experiment with anti-TNF antibody (Fig 5A) and was found to be 1 focus-forming unit (FFU) in 5 μL medium. This equates to adding virus at less than MOI 0.0001 in that experiment. This together with the lack of effect of DENV at up to MOI 5 (Fig 5C) indicates lack of a detectable role for DENV particles in the monolayer disturbance in the time course studied. In summary, the ability of anti-TNF to almost completely abrogate the effect of the medium from DENV-infected GM-CSF macrophages confirmed that TNF was the major mediator of endothelial permeability.

It is also noteworthy that the DENV-infected media reliably mediated an initial increase in the cell index that peaked 1 h after addition to HUVEC monolayers (Figs 4A–C and 5A) and was followed by a decline with nadir between 4–5 h. The increase in cell index was not observed with the medium from the mock-infected cells (Fig 4A and 4B) or the virus alone (Fig 4C). The increase in cell index with DENV-infected medium was partially diminished upon treatment with the TNF antibody (Fig 5A) and observed to a small extent with TNF alone (Fig 4A and 4B), consistent with a contribution from TNF. However, it is unclear what other components of the DENV-infected media contribute to this effect.

## Discussion

In view of the increasing incidence of DENV infections and concomitant rise in severe cases, an understanding of the factors that drive severe dengue disease is important to enable development of specific treatments. Amongst the wide range of molecules proposed to mediate pathogenesis of severe DENV infection, the virus-derived NS1 protein is suggested to contribute to disease via multiple mechanisms including induction of proinflammatory cytokines via TLR4 activation, mediation of vascular leak through effects on the glycocalyx, and inhibition of complement, which may enable the virus to evade the immune system [21,23,33,35]. Here we showed that DENV2 NS1 produced as a recombinant protein by mammalian HEK293 or CHO-S cells or produced by DENV2-infected Vero or K562 cells did not activate TLR4, which is contrary to some previous studies [21,33,35]. Furthermore, we found that purified recombinant NS1 used at biologically relevant levels only had a minor effect on endothelial monolayer integrity when compared to effects of LPS or TNF, with the latter also used at a concentration relevant to disease [46–49]. Finally, we showed that TNF was the predominant cause of endothelial monolayer disruption in medium from DENV-infected human macrophages, which contained relatively low levels of NS1.

NS1 was previously reported as an activator of TLR4 that induces a proinflammatory response based on studies showing TLR4 activation by recombinant NS1 [21,33,35]. Those studies used purified insect cell-expressed untagged NS1 at ≥ 5 μg/mL [21,35], His-tagged NS1 at 50 μg/mL [33] or concentrated medium from CHO-S cell expression of untagged NS1 at 5 μg/mL [21]. Here, we tested TLR4 activation by recombinant untagged NS1 in expression medium from HEK293 or CHO-S cells, which was characterised as being structurally sound and by NS1 in medium from DENV-infected cells, which is likely to resemble the form that circulates in disease. Media were minimally processed to prevent contamination with LPS. NS1 concentrations used were equivalent to or higher than that of untagged NS1 used in previous studies [21,35] and higher than the levels in the majority of severe dengue patients reported in one study, which ranged from 0.6 to 2.5 μg/mL [24]. NS1 did not activate TLR4 and there was nothing in the NS1 samples that was interfering with TLR4 activation by LPS. One limitation of our study is that we have used NS1 from a single DENV2 strain, ET300. However, we note that it has the same sequence as the NS1 used in a previous study, where NS1 was shown to activate TLR4 and disrupt endothelial monolayer integrity [21]. Thus, the discrepancy between our results and previous work is unlikely to be due to a difference in the NS1 sequence. Consequently, we conclude that the protein component of the NS1 lipoprotein does not stimulate TLR4, and it is possible that prior results are due to it carrying an inflammatory host-derived lipid or LPS itself. Furthermore, the NS1 did not potentiate the TLR4 response to LPS making this unlikely to be a contributory function for NS1 in dengue pathology. Notably, two recent studies showed that NS1 became marginally proinflammatory upon incubation with HDL [34] or LDL [50]. However, some results with HDL were not reproduced [50]. In agreement with our finding, these studies showed that His-tagged NS1 purified from insect cells or HEK293 cells was not proinflammatory on human monocyte derived macrophages at 10 μg/mL or 2 μg/mL, respectively [34,50].

Over the years numerous self and foreign proteins have been suggested as TLR4 ligands [51–53] but such studies are fraught with problems of ensuring lack of LPS contamination, and it has been suggested that these reported protein ligands are either binding to LPS or sensitising cells to low amounts of LPS [54]. Detection of LPS is imperfect. Previous studies on NS1 tested for LPS [21,33,35] using the *Limulus* amebocyte lysate (LAL) assay that is based on the ability of the horseshoe crab factor C protein to bind LPS and become proteolytically active [55]. However, in the presence of proteins with lipid-binding/LPS-binding activity, the results will depend on how efficiently LPS can be extracted by factor C from the protein [56]. In cell-based assays to measure TLR4 activation, LPS binding protein (LBP) and soluble CD14 are present in the medium and can aid in extraction of tightly bound LPS. Furthermore, it has been reported that LPS binds to poly-His tags [57] and LPS quantification in purified His-tagged proteins has been noted to be inaccurate using the LAL assay [56]. Although TLR4 activation by NS1 was shown to be insensitive to the LPS-binding antibiotic polymyxin B [21,33], polymyxin B, like factor C, would have to compete with LPS-binding proteins. Having now shown that recombinant NS1 protein does not activate TLR4, we suggest that a cell-based TLR4 activation assay is also used in future studies with NS1 to assess for the presence of LPS or other TLR4 activating lipids.

The lack of activation of TLR4 by NS1 does not exclude the reported role for TLR4 in dengue infection [21]. Mouse models of infection with a number of viruses have been reported to show TLR4-dependent pathology defined using either TLR4 knockout mice or inhibitors, including respiratory syncytial virus (RSV), influenza virus, Japanese encephalitis virus, Ebola virus and Marburg virus [58–61]. Although a role for various viral proteins including SARS-CoV-2 spike protein in activating TLR4 has been suggested [53], in the case of the spike protein, a subsequent study showed that the LPS-free mammalian cell-expressed protein did not have proinflammatory activity [62]. For the other viral proteins, including Ebola virus glycoprotein and RSV fusion protein, it is not clear whether responses are mediated by these proteins or bound inflammatory lipids. A role for TLR4 in pathology of mouse models of viral infection could also be the result of LPS influx following gut epithelial barrier breakdown.

Purified His-tagged NS1 at 16–18 μg/mL compromised HUVEC monolayer integrity when compared to His-tagged albumin that was expressed and purified in parallel. However, the effect was minor when compared to 100 ng/mL LPS or 0.1 ng/mL TNF, in contrast to previous studies [21,22]. Notably, we used the xCELLigence RTCA instrument that measures electrical impedance, which has been used in several studies to assess effects on endothelial barrier integrity [39–43]. Previous studies with recombinant untagged or His-tagged NS1 used the EVOM2 resistance meter, which is designed to measure trans-epithelial electrical resistance [21,22]. The EVOM2 manufacturer recommends the commonly used chopstick electrodes for epithelial but not endothelial studies due to the much lower resistance of the endothelial monolayers, for which an Endohm chamber is recommended. The advantage of the xCELLigence instrument is that the cells are grown on plates with interdigitated gold microelectrodes on the bottom of the well that enable continual measurement of electrical impedance without removal of the plated cells from the incubator.

A recent study showed that a peptide mimetic of apolipoprotein (apo) A-I, the major protein of HDL, inhibited the effect of NS1 on endothelial barrier integrity *in vitro* and promoted survival of dengue-infected mice [63]. The apo A-I peptide mimetic can bind LPS and has been shown to protect against LPS-induced inflammation [64]. Furthermore, although an unrelated study showed that apo A-I inhibited NS1 activation of TLR4 [33], our data indicates that NS1 protein does not activate TLR4. Thus, the apo A-I may have inhibited activation of TLR4 by contaminating LPS in the purified NS1 [33]. Finally, the apo A-I peptide mimetic has been shown to have numerous beneficial effects against atherosclerotic vascular disease and other chronic inflammatory disorders [65], reduced infectivity of HIV [66] and HSV [67] and inhibited SARS CoV2 replication *in vitro* [68] but how these varied responses are mediated is unknown. Consequently, the route through which apo A-I mimetic mediates improved survival of dengue-infected mice [63] is unclear.

The role of NS1 in severe disease remains enigmatic. In support of a role for NS1 several studies have shown antibodies against NS1 protecting against severe dengue disease in mouse models [6,20,69,70]. However, a further study failed to demonstrate protection by NS1-targetting antibodies, and it was suggested that the pathogenic role of NS1 is strain-dependent [71]. When protection with NS1 antibodies is observed, there could be several effects apart from interference with NS1 interaction with endothelial cells. Antibodies targeting NS1 on the surface of virus-infected cells may induce antibody-dependent cellular cytotoxicity [72,73]. Additionally, since NS1 has been implicated in immune evasion via its ability to inhibit complement [74–76], NS1 antibodies may prevent the ability of NS1 to inhibit complement-mediated lysis of the virus [2,23].

TNF was the key mediator of endothelial monolayer permeability released by DENV-infected GM-CSF macrophages in this study. The macrophages released high levels of TNF, up to 8 ng/mL, consistent with a previous study [45], but modest levels of NS1 after 2 days of infection (~500 ng/mL). A blocking antibody to TNF mostly ablated the effect of medium from the infected cells on endothelial permeability. Our data agree with the key role of TNF indicated by protection from severe disease in mouse models by blocking antibodies to TNF [8–10]. The role of TNF in DENV-infected patients is less clear. While some studies indicate a correlation between TNF and severe DENV infection [13–15] other studies do not [16]. The lack of consistency is suggested to be due to timing of sample collection and processing, study design and population differences, as well as a contribution from molecules other than TNF [4]. Also noteworthy is that the concentration of TNF in severe DENV patients from different studies is quite variable with levels from 50-60 pg/mL [46,47] to levels 10-fold higher [48] and even up to 922 pg/mL in the acute phase [49]. We observed a substantial effect on endothelial permeability with 100 pg/mL TNF, which is within the range observed in severe DENV patients indicating that TNF is likely to contribute to vascular leak in disease. However, the effect of TNF can be modulated by other factors. For example, it has been shown that type I IFN can inhibit the effect of TNF on endothelial permeability [77]. The presence of such modulatory factors may explain why TNF levels alone may not be predictive of severe disease.

In summary, we have shown that NS1 produced under stringent sterile and LPS-free conditions does not activate TLR4 and that it only has a modest effect on endothelial monolayer integrity compared to TNF and LPS. Although we found that TNF was the major mediator of endothelial permeability released from infected GM-CSF macrophages, we acknowledge that the concentration of NS1 was low in these samples, and we do not exclude that NS1 in patients may have some TLR4-independent effects on endothelial cells. Furthermore, the disease pathology *in vivo* is more complex involving many more inflammatory mediators and modulators [2,7,77]. Future experiments with serum from patients with severe dengue disease may give further insights into the key mediators and modulators associated with vascular leak.

## Materials and methods

### Ethics statement

Work with human blood was approved under the University of Queensland Institutional Human Research Ethics Approval (2015000123). Buffy coats from anonymous donors were obtained from the Australian Red Cross Lifeblood Service under agreement deed 23–08QLD-15. Formal consent was not obtained from donors due to anonymity.

### Expression and purification of recombinant proteins

All handling of the cultures or media was under sterile conditions. Plasmids expressing untagged or C-terminally His-tagged NS1, from DENV serotype 2 ET300 strain (Genbank accession number MT921572.1), with the N-terminal signal sequence of a murine IgG heavy chain were kindly provided by Prof. Daniel Watterson and Dr. Naphak Modhiran (University of Queensland, Brisbane, Australia). The sequence of the expressed proteins is shown in S1 Fig. His-tagged albumin was cloned into the same plasmid backbone for expression with the same signal sequence. Plasmids were purified using an endofree plasmid purification kit (Qiagen). Suspension HEK293 (Expi293F) and CHO-S cells (both from Thermo Fisher Scientific) were cultured in Expi293 or CD CHO expression medium respectively, with 100 U/mL penicillin and 100 μg/mL streptomycin (all from Gibco, Thermo Fisher Scientific). Cultures were maintained at a density of 0.2-3 × 10^6^ cells/mL and transfection was with PEI (Polysciences). Transfection complexes were prepared with a 1:4 or 1:6 w/w ratio of DNA to PEI. CHO-S expression cultures were supplemented with 7.5% CHO CD Efficient feed A and B (Gibco, Gibco, Thermo Fisher Scientific). Expression medium was harvested on day 4 following transfection and filtered through a 22 μm PES filter (Merck Millipore). Where stated, medium was concentrated using an Amicon 10 kDa cut-off centrifugal filter unit (Merck Millipore). His-tagged proteins expressed in parallel using suspension HEK293 were batch purified on nickel fast flow affinity resin (Cytiva) and eluted using imidazole elution buffer. The eluted proteins were filtered through a 0.22 μm filter, buffer exchanged into PBS and concentrated using an Amicon 10 kDa cut-off centrifugal filter unit, filtered through a 0.2 μm PES filter (Cytiva) and quantified with a BCA protein assay (Sigma) using 2 mg/mL BSA standard (Thermo Fisher Scientific).

### Treatment of HEK-Blue hTLR4 cells

The HEK-Blue hTLR4 cell line (Invivogen) is engineered to express human TLR4, MD2 and CD14 and contains all the other components of the TLR4 signalling pathway required for NF-κB activation as well as an NF-κB-driven secreted embryonic alkaline phosphatase (SEAP) reporter to assess TLR4 activation. Cells were grown in complete DMEM with 4.5 g/L glucose, 110 mg/L sodium pyruvate, supplemented with Glutamax, 10% heat inactivated fetal bovine serum (HI-FBS), 100 U/mL penicillin and 100 μg/mL streptomycin (all from Gibco, ThermoFisher Scientific). Cells were maintained in a humidified incubator at 37^o^C with 5% CO_2_. To test TLR4 activation, cells were plated in 96-well plates (50 000 cells per well in 50 μL) and incubated with 40 μL of neat or concentrated NS1 expression medium or empty vector medium and then 10 μL of DMEM medium or LPS (ultrapure LPS from *Escherichia coli* 0111:B4 strain, Invivogen) diluted in DMEM medium was added and the cells incubated for 18 h. To test the medium for secreted embryonic alkaline phosphatase (SEAP), 5 μL of medium was transferred to a 394 well plate and then 45 μL of QUANTI-blue solution (Invivogen) was added. The plate was incubated at 37^o^C and the absorbance at 640 nm was measured after 60 min using a Clariostar microplate reader (BMG Labtech).

### Treatment of peripheral blood mononuclear cells (PBMCs)

Human PBMCs were prepared from buffy coats by density gradient centrifugation on Ficoll-Paque. NS1 expression medium or empty vector medium were buffer exchanged into PBS on a 10 kDa cut-off centrifugal filter unit and diluted to various concentrations in complete RPMI with Glutamax, 10% HI-FBS, 100 U/mL penicillin and 100 μg/mL streptomycin (all from Gibco, Thermo Fisher Scientific). PBMCs in complete RPMI medium were plated in 96-well plates (100,000 per well in 50 μL) and 40 μL of buffer-exchanged NS1 or empty vector medium and 10 μL of RPMI medium or LPS diluted in RPMI medium was added and the cells incubated for 18 h and the medium analysed using an IL-6 ELISA assay (R&D systems).

### Preparation of virus stock

DENV2 ET300 strain was kindly provided by Prof. Paul Young (University of Queensland, Brisbane, Australia) and was grown in C6/36 cells and purified as described [19]. Viral titers were determined in Vero cells using a fluorescent immuno-plaque assay [19].

### Production of NS1 from DENV2-infected Vero and K562 cells

Vero cells were maintained in complete DMEM while K562 cells were maintained in complete RPMI. For infection of Vero, 3 × 10^6^ cells were plated in 15 mL of DMEM infection medium, which is similar to complete DMEM but with 2% HI-FBS and 25 mM HEPES, and then infected at MOI 4. For K562, 1.2 × 10^6^ cells were first plated in 4 mL of serum-free RPMI, with Glutamax, 100 U/mL penicillin, 100 μg/mL streptomycin and 25 mM HEPES, and incubated with virus at MOI 37 for 1 h. Then the medium was adjusted to 2% HI-FBS in a final volume of 6 mL. For both cell lines, media were harvested on day 4, replaced with fresh medium and then harvested again on day 7. Harvested media were centrifuged at 9000 *g* to pellet debris and then buffer exchanged into PBS and concentrated approximately 10-fold using an Amicon 100 kDa cut-off centrifugal filter unit. The level of NS1 in the concentrated media was measured by ELISA and the media tested for TLR4 activation in HEK-Blue hTLR4 cells.

### Preparation and infection of GM-CSF-differentiated monocytes

Monocytes were isolated from 20 or 40 million PBMC (prepared as above) using CD14 microbeads (Miltenyi Biotec) and cultured in 10 mL of complete RPMI with 10 ng/mL GM-CSF. The medium was changed on day 6 and the GM-CSF macrophages harvested on day 7. The cells were resuspended in RPMI without FBS, plated at 90,000 cells per well in 100 μL and incubated with DENV2 at MOI 20 or with an equivalent volume of PBS (mock infection) for 90 min and then 70 μL medium with HI-FBS and GM-CSF added so that the final concentration was 10% HI-FBS and 10 ng/mL GM-CSF. The cells were incubated for a further 2 days and the medium harvested.

### Size exclusion chromatography (SEC)

Samples (0.5 mL) were analysed by SEC using a Superdex 200 increase 10/300 GL column (Cytiva), which was run on an AKTA pure FPLC (Cytiva) in PBS at 0.75 mL per min and 0.25 mL fractions were collected. Assessment of molecular weight ranges of fractions was provided by a calibration curve supplied with the column and validated with mammalian cell-expressed and purified human albumin and soluble CD14 proteins.

### NS1 ELISA

NS1 concentration was determined by indirect sandwich ELISA. Capture antibody was GUS2 antibody [78] while detection antibody was DV2 CMP2 polyclonal antibody (Panbio) followed by anti-rabbit-HRP (Cell Signaling Technology). TMB substrate (Sigma) was added, and the reaction terminated with 2M sulphuric acid.

### TNF assay

TNF was assayed using the AlphaLISA High Performance (HP) Human TNFα Detection Kit (Revvity).

### Negative stain electron microscopy

Fractions 22–25 from SEC of the concentrated NS1 medium were pooled and concentrated 20-fold on an Amicon 100 kDa cut-off centrifugal filter unit (Merck Millipore). Carbon-coated grids (EMS) were glow-discharged for 85 s before 4 μL of NS1 protein (10 μg/ml) was applied to the grid and allowed to incubate for 2 min. The grids were rinsed twice with 20 μL of water and stained with 2% Uranyl Acetate. Micrographs of NS1 were acquired using a Hitachi 7700 operated at 120 kV.

### Measurement of HUVEC monolayer integrity

Pooled human umbilical vein endothelial cells (HUVEC) (Lonza) were cultured in EGM-2 medium (HUVEC medium) and used at less than passage 8 unless stated otherwise. To study effects on endothelial monolayer integrity the xCELLigence Real-Time Cell Analysis (RTCA) instrument (Roche/ACEA Biosciences) housed in a 5% CO_2_ incubator at 37°C was used. Cells were plated in a 96-well E-plate view (Agilent) at a density of 6000 cells per well in 200 μL. Unless stated otherwise the electrical resistance, expressed as cell index, was recorded every 15 min and the cells were grown for 3 days, with 140–200 µL of medium changed daily, to allow formation of a monolayer with stable cell index. On day 3, 100 μL of medium was removed and replaced with an appropriate volume so that the final volume after adding samples would be 200 μL. The plates were then incubated for 6 h prior to sample addition to allow the cell index to stabilise. Upon sample addition, the plate was incubated for a further 18 h. For data analysis, the cell index for all wells was normalised at the time point just before sample addition and data is presented as the normalised cell index.

### Neutralisation of TNF with Anti-TNF-α antibody

Anti-TNF antibody clone D2E7 (Thermo Fisher Scientific) is a non-therapeutic biosimilar antibody to the therapeutic antibody adalimumab. The anti-TNF at 5 mg/mL in PBS was diluted to 1 mg/mL in HUVEC medium. Media from DENV (DV) or mock-infected cells were incubated on ice for 1 h with antibody at a final concentration of 100 μg/mL or with an equivalent volume of HUVEC medium. The samples were then diluted ten-fold into a 96-well E-plate with HUVEC monolayers.

## Supporting information

S1 Fig Sequence of the His-Tagged NS1 protein with the N-terminal signal sequence expressed from the pNBF plasmid. (TIF)

S1 Table Combined raw data (Excel file). (XLSX)
