# Supplementary figures and images for: Dengue virus NS1 protein does not activate TLR4 and has a modest effect on endothelial monolayer integrity compared to TNF
Source: PLoS Pathog. 2025 Nov 11;21(11):e1013695. doi: 10.1371/journal.ppat.1013695 (PMC12622831; doi:10.1371/journal.ppat.1013695)

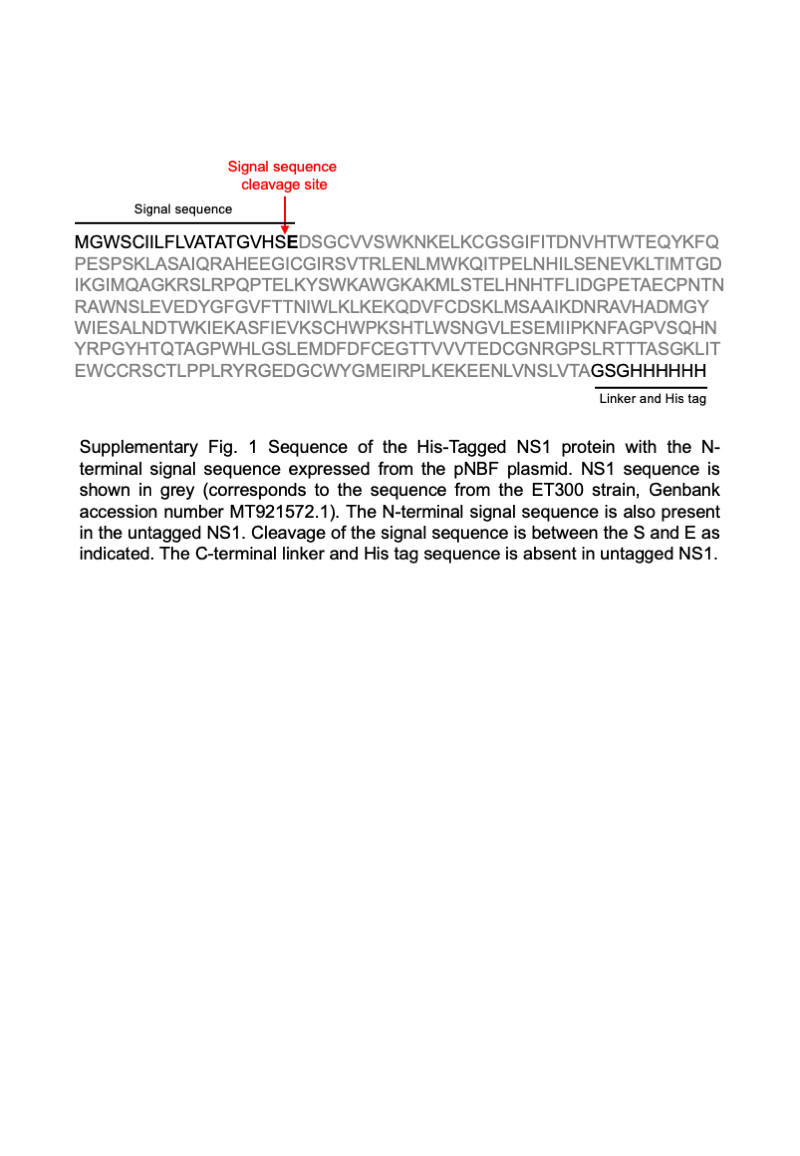

Supplement: S1 Fig — (TIF) [file ppat.1013695.s001.tif]
